# Risk factors for persistent fatal opioid-involved overdose clusters in Massachusetts 2011–2021: a spatial statistical analysis with socio-economic, accessibility, and prescription factors

**DOI:** 10.1186/s12889-024-19399-5

**Published:** 2024-07-15

**Authors:** Sumeeta Srinivasan, Jennifer Pustz, Elizabeth Marsh, Leonard D. Young, Thomas J. Stopka

**Affiliations:** 1 Department of Urban and Environmental Policy and Planning, Tufts University, Medford, MA USA; 2 Department of Public Health and Community Medicine, Tufts University School of Medicine, Boston, MA USA; 3 Institute for Health Metrics and Evaluation, Seattle, WA USA; 4 Prescription Monitoring Program, Massachusetts Department of Public Health, Boston, MA USA

**Keywords:** Opioids, Local Indicators of Spatial Association (LISA) clusters, Empirical bayes smoothing, Empirical bayes LISA, Opioid death, Overdose, Social determinants of health, Public health, Built environment, Spatial statistics

## Abstract

**Background:**

Fatal opioid-involved overdose rates increased precipitously from 5.0 per 100,000 population to 33.5 in Massachusetts between 1999 and 2022.

**Methods:**

We used spatial rate smoothing techniques to identify persistent opioid overdose-involved fatality clusters at the ZIP Code Tabulation Area (ZCTA) level. Rate smoothing techniques were employed to identify locations of high fatal opioid overdose rates where population counts were low. In Massachusetts, this included areas with both sparse data and low population density. We used Local Indicators of Spatial Association (LISA) cluster analyses with the raw incidence rates, and the Empirical Bayes smoothed rates to identify clusters from 2011 to 2021. We also estimated Empirical Bayes LISA cluster estimates to identify clusters during the same period. We constructed measures of the socio-built environment and potentially inappropriate prescribing using principal components analysis. The resulting measures were used as covariates in Conditional Autoregressive Bayesian models that acknowledge spatial autocorrelation to predict both, if a ZCTA was part of an opioid-involved cluster for fatal overdose rates, as well as the number of times that it was part of a cluster of high incidence rates.

**Results:**

LISA clusters for smoothed data were able to identify whether a ZCTA was part of a opioid involved fatality incidence cluster earlier in the study period, when compared to LISA clusters based on raw rates. PCA helped in identifying unique socio-environmental factors, such as minoritized populations and poverty, potentially inappropriate prescribing, access to amenities, and rurality by combining socioeconomic, built environment and prescription variables that were highly correlated with each other. In all models except for those that used raw rates to estimate whether a ZCTA was part of a high fatality cluster, opioid overdose fatality clusters in Massachusetts had high percentages of Black and Hispanic residents, and households experiencing poverty. The models that were fitted on Empirical Bayes LISA identified this phenomenon earlier in the study period than the raw rate LISA. However, all the models identified minoritized populations and poverty as significant factors in predicting the persistence of a ZCTA being part of a high opioid overdose cluster during this time period.

**Conclusion:**

Conducting spatially robust analyses may help inform policies to identify community-level risks for opioid-involved overdose deaths sooner than depending on raw incidence rates alone. The results can help inform policy makers and planners about locations of persistent risk.

## Introduction

The opioid overdose crisis has persisted as one of the most significant public health challenges of the past two decades in the US. Between 1999 and 2019, the national age-adjusted opioid-involved overdose rate increased from 2.9 per 100,000 population to 15.5 per 100,000 [1]. Rates continued to increase steeply during the COVID-19 pandemic [2]. The rate climbed to 21.4 per 100,000 during 2020 and then to 24.7 per 100,000 in 2021 [3]. Commonly understood drivers of the overdose crisis include drug supply and demand-side pressures. Supply-side drivers have evolved over multiple waves, from prescription opioids, to heroin, to illicitly manufactured fentanyl [4, 5]. Demand-side drivers include deindustrialization and concentrated poverty, pain arising from work-related injuries, [5] income inequality, [6] and added stress, isolation, and economic disadvantage connected to the COVID-19 pandemic [7, 8]. 

More studies have begun to consider the association between drug use, opioid-related mortality, and the built environment, defined by Ezell and colleagues as “the purposeful creation and spatial arrangement of housing, sidewalks, roadways, retail and institutional buildings, public transit, and green spaces.” [9–11] Research has already suggested that the built environment influences health and health behaviors [12], including substance use [9, 10, 13] and opioid-related mortality [11]. For example, analgesic opioid-involved overdose fatalities were found to be more likely to occur in “fragmented” neighborhoods than in higher-income neighborhoods in New York City [14]. Chichester et al. found that bus stops and public schools were associated with increased risk of opioid overdose in rural areas of an Alabama county and that inpatient treatment centers, transitional living facilities, express loan establishments, and liquor vendors were associated with increased opioid overdose risk in urban areas of the same county [15]. Inequality and racial and ethnic composition of neighborhoods have also been identified as correlates of increased opioid mortality [16, 17]. 

The connection between substance use and built environment variables (access to public restrooms, access to pharmacies, and driving distance to services, defined in our study as fast-food restaurants, gas stations, and highway exits) is also important. Prior studies found that people who inject drugs often use drugs in public restrooms [9, 18, 19]. Pharmacies represent an important access variable for several reasons. During the initial wave of the overdose crisis, pharmaceutical prescriptions, either legitimate, diverted, or potentially inappropriate, fed the opioid supply [4, 17, 20]. In addition, naloxone (a medication to reverse an opioid overdose) is available at pharmacies without a prescription, [21, 22] although this provision may vary by neighborhood socio-demographic levels [23]. For example, prescription opioid poisoning increased more in postal codes with greater pharmacy density in California in the time period between 2001 and 2010 [24]. Road access to services may mediate several factors related to substance use, such as access to meeting places to buy illicit substances, as well as access to harm reduction services including syringe services programs [9]. 

Over the past two decades, Massachusetts’ opioid-involved mortality rate has often been higher than the national rate and, at times, twice as high [25]. The most recent data released by the Massachusetts Department of Public Health indicated that opioid-involved mortality rate peaked at 33.5 per 100,000 population in 2022 [26]. Studies focusing on the overdose crisis in Massachusetts have explored potential intersections between the built environment and opioid overdoses. A spatial analysis of potentially inappropriate opioid prescribing (PIP) identified several overdose and PIP clusters, but did not find a significant overlap between the two [20]. Other studies identified a rural county in Massachusetts with both good access to harm reduction services but persistently high overdose rates, [27] and that a majority of overdoses in the state occurred at home between 2015 and 2017 [28]. A recent study of opioid-related deaths in Massachusetts incorporated psychosocial, economic, built environment, and health-related variables using multilevel mixed-effects regression models, found that none of the built environment variables had a statistically significant association with opioid-related mortality [29]. 

While these studies across the US and within Massachusetts have contributed importantly to our understanding of the relationships between substance use and the built environment, many often fail to use spatial statistical methods that properly account for excess zero counts for overdose outcomes and spatial autocorrelation, two of the most common confounding factors in spatial epidemiology [30]. With spatial analysis of rare events, such as overdose deaths, the distribution of death counts is often “zero-inflated” (i.e., a large number of locations have no deaths while a few locations have many). Several methods have been used to address this issue, including use of: small area estimation techniques; [31] a rate smoothing technique to examine characteristics of prescription opioid poisoning; smoothing rates in the ZIP Code Tabulation Area (ZCTA) with small populations; and Empirical Bayes smoothing. [32–35] These methods include data from adjacent spatial units that enable refined estimates for all locations, but are of elevated importance in locations that have low population density such as rural areas in western Massachusetts. Two models that acknowledge spatial autocorrelation and account for excess zeros include zero-inflated Poisson regression models, [36, 37] such as the Besag-York-Mollie model, [38] and a Bayesian hierarchical space–time misalignment Poisson model [24, 39]. 

The problem of excess zeros masking potentially non-zero true counts and rates exists in Massachusetts analyses because the geographical distribution of opioid overdose deaths in Massachusetts spans urban, suburban, and sparsely populated rural areas that are likely to have very low or zero counts. To date, we are only aware of a few studies of opioid-involved overdose deaths using Massachusetts data that have employed techniques that acknowledge zero-inflation and spatial autocorrelation at smaller spatial units (such as the ZCTA level), [40] but these techniques could greatly help in enhancing our understanding of geographic variation in overdose rates across Massachusetts.

**Fig. 1:**
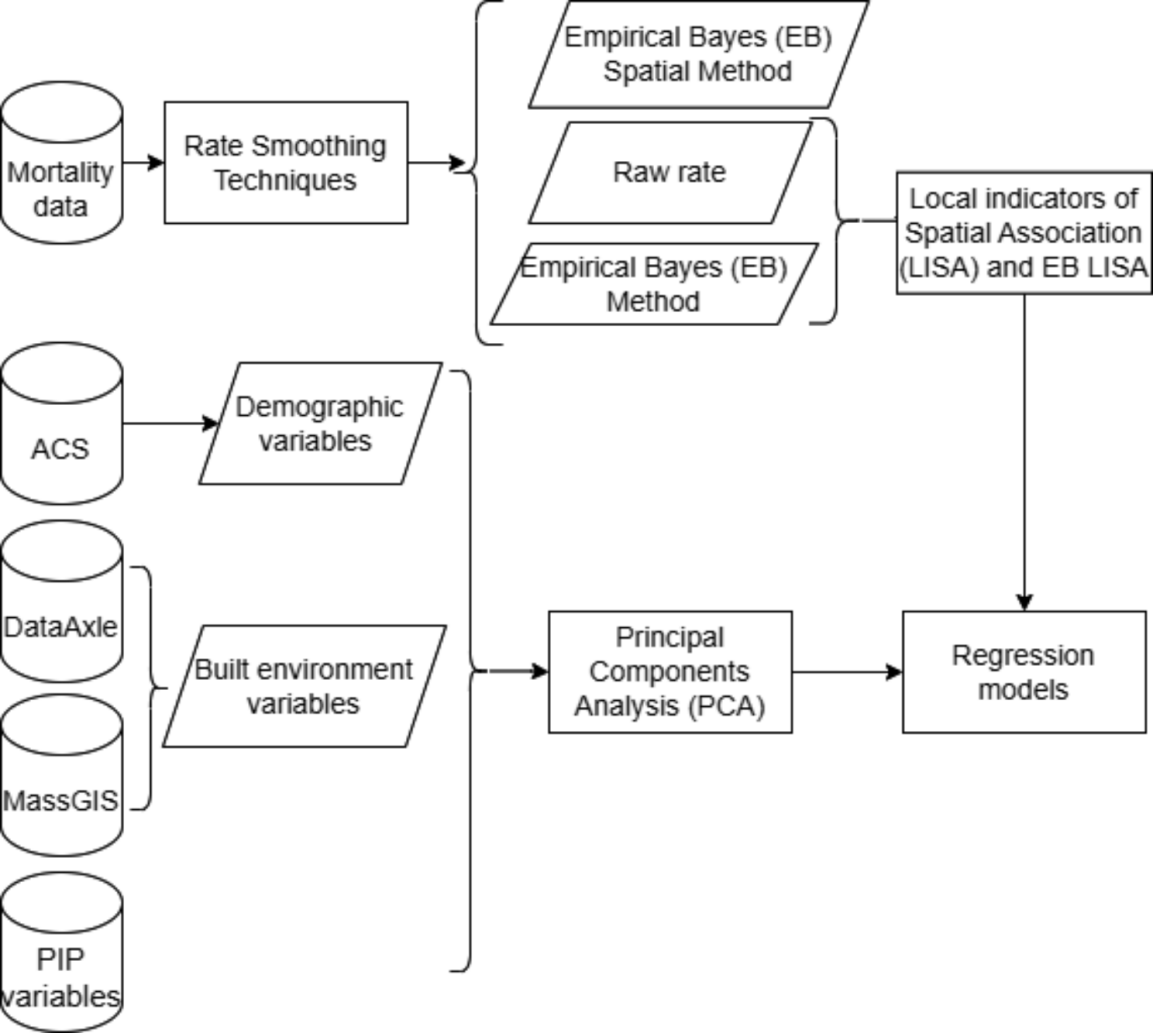
Flow diagram visualizing data and methods described in this paper. Note: PIP is potentially inappropriate opioid prescribing. ACS represents data from the US Census Bureau’s American Community Survey. Data Axle was used for built environmental variables such as gas stations and pharmacies; MassGIS is the Massachusetts State GIS Data provider; LISA (Local Indicators of Spatial Association) statistics generate clusters

The goal of our study was to identify fatal overdose clusters as well as socioeconomic and built environment factors associated with opioid-related overdose rates in Massachusetts from 2011 to 2021 while acknowledging spatial autocorrelation. We aimed to: (1) address the issue of zero-inflated opioid-involved overdose rates by comparing three spatial methods (raw rates, Empirical Bayes, and Empirical Bayes Spatial); (2) utilize the raw and Empirical Bayes rate as well as Empirical Bayes Local Indicator of Spatial Autocorrelation (LISA) to identify statistically significant fatal opioid overdose clusters in Massachusetts between 2011 and 2021; (3) derive socio-environmental and pharmacological variables using PCA in those clusters; and (4) model community-level factors associated with overdose rates using Conditional Autoregressive Empirical Bayes logistic regression models to predict if a ZCTA was part of a cluster and Conditional Autoregressive Empirical Bayes zero inflated Poisson regression models to predict how many times it was part of a cluster during the time period while acknowledging spatial autocorrelation of the outcome (Fig. 1).

### Data

#### Data sources

We obtained fatal opioid-related overdose data by address of residence for decedents between 2011 and 2021 from the Massachusetts Registry of Vital Records and Statistics (RVRS) [41]. These data included sociodemographic characteristics of decedents, including sex, race, ethnicity, and age at the time of death. We obtained opioid prescription data from the Massachusetts Prescription Monitoring Program (MA PMP), aggregated at the zip code or ZCTA level [42]. We obtained address level data for services (gas stations, fast food restaurants, pharmacies), and “access to infrastructure” measures (highway exits, major roads) from Data Axle and from MassGIS (Massachusetts Bureau of Geographic Information) [43, 44]. We obtained pharmacy addresses from the Massachusetts Board of Registration in Pharmacy [22]. Finally, we compiled population level sociodemographic data at the ZCTA level, from the US Census Bureau’s American Community Survey (ACS), relying on 2011 to 2015 and the 2017 to 2021 5-year estimates for people aged ≥ 10 years for calculating rates [45]. We used ACS 2011–2015 data for the years 2011–2016 and 2017–2021 data for the years 2017–2021.

#### Outcome

The two outcomes were whether a ZCTA was part of a cluster or not and the number of times a ZCTA was within a fatal overdose cluster based on annual opioid-related overdose rates between 2011 and 2021. We calculated the LISA statistic for each year between 2011 and 2021 for the raw rates, and for Empirical Bayes smoothed rates. We also calculated the Empirical Bayes LISA for the same time period, as this adjusts for variation in the underlying population. [46, 47] We did not use the spatial EB rates for calculating clusters. This is because spatial EB rates were useful to identify locations at risk, but they also resulted in over-smoothing of the rates.

#### Covariates

PIP measures were aggregated by ZCTA. We defined PIP measures, established through our previous research, for the years 2011–2017 at the ZCTA scale: high Morphine Milligram Equivalents or MME ( > = 90), co-prescribing of benzodiazepines and opioids, poly-pharmacy opioid prescription fills, multiple provider episodes (i.e., doctor shopping), >=3 cash purchases of opioid prescriptions and opioid prescriptions without a pain diagnosis. We were not able to obtain PIP data for the years 2018–2021.

#### Socioeconomic measures

We selected socioeconomic measures from the ACS at the ZCTA-level based on the literature and our previous research [20, 48, 49]. Poverty as a percentage of households living below the poverty threshold was defined by the five-year ACS estimates. We also included median age, and population percentages by race and ethnicity for non-Hispanic White, Black, Asian, American Indian and Alaskan Native, and Hispanic communities.

#### Built environment measures

We compiled built environment measures based on the literature and our previous research [20, 48, 49]. Specifically, we selected gas stations and fast-food restaurant locations as a proxy for access to public restrooms, locations where overdoses often occur [18, 19, 43, 50, 51]. We used pharmacy addresses to analyze the spatial distribution of access to sources of over-the-counter naloxone [22]. We compiled highway exits and major roads from a Massachusetts GIS agency (MassGIS) as a proxy for access to services. For each ZCTA we calculated the distance to the nearest exit, major road, pharmacy, fast food restaurant, and gas station from the centroid of the ZCTA. We also calculated the average gas station density for each ZCTA.

## Methods

Figure 1 provides an overview of the data and methods used in this paper.

### Death rate mapping

Using ArcGIS Pro 3.1 (ESRI, Redlands, CA) we created maps of raw and Empirical Bayes and Empirical Bayes Spatial smoothed opioid overdose death rates at the ZCTA level across the state.

### Spatial smoothing techniques

Smoothing techniques work by detrending the overdose rate within target polygons (ZCTAs) and by using data from neighboring polygons, thus allowing for the calculation of a local average overdose measure that is less susceptible to variation due to outlier values. We employed two spatial smoothing techniques to derive stable estimates for spatial measures (fatal overdose rates). These included: (1) Empirical Bayes Method; and, (2) Empirical Bayes Spatial method [52–54]. 

The Empirical Bayes smoothing for rate calculations relies on calculating a weighted average of the raw rate for each ZCTA and the state average, with weights proportional to the underlying population at risk. Therefore, ZCTAs with a small population at risk will tend to have their rates adjusted considerably, while rates for ZCTAs with larger populations at risk will not change much. The second method was Empirical Bayes Spatial smoothing, which is like Empirical Bayes smoothing, except that the reference rate is computed for a group of neighboring ZCTAs that share a boundary with each individual ZCTA, rather than taking the same overall reference rate for all ZCTAs. We used the R package rgeoda to calculate Empirical Bayes and Empirical Bayes Spatial smoothed rates [53, 55]. We then used the LISA statistic to identify and map clusters (i.e., ZCTAs with high overdose rates surrounded by ZCTAs with high rates or high-high (HH) locations and ZCTAs with high rates surrounded by low rates or high-low (HL) locations for (i) the raw rates, (ii) the Empirical Bayes smoothed rate and (iii) using the Empirical Bayes LISA method [47, 53]. We did not calculate LISA for Empirical Bayes Spatial rates to avoid over-smoothing the data. Clusters were considered statistically significant if the local pseudo-p value *p* < 0.05 as estimated by rgeoda. LISA were also calculated using the rgeoda package. We used the queen’s contiguity criterion to define all the spatial weights matrix calculations.

### Principal components analysis (PCA)

To avoid multicollinearity amongst the PIP, access, and socioeconomic variables due to significant cross correlation, we used PCA with a Varimax rotation to extract new variables that summarized the covariance in the PIP, socioeconomic, and access variables. PCA is a dimension reduction technique that is commonly used to reduce the number of variables used in analyses while preserving the information from them. We used a cut point of 0.25 for the included factor loadings as recommended in other studies [56]. The psych and dplyr packages in R were used for PCA and data management [57].

#### Statistical modeling

We fit three Bayesian logistic regression models where the dichotomous outcome characterized whether a ZCTA was in a HH or HL cluster or not (0/1), as identified by (i) the raw overdose rate LISA, (ii) the Empirical Bayes rate LISA and (iii) the Empirical Bayes-corrected LISA developed by Assuncao and Reis [47]. The components derived from the PCA were the explanatory variables for all three models. Models fit with all the variables (without PCA) resulted in very high VIF values due to multicollinearity. We used Conditional Autoregressive (CAR) Bayes regression models to fit both the logistic regression model focused on whether the ZCTA was in a cluster for individual years between 2011 and 2021, as well as a zero-inflated Poisson regression model for the number of times the ZCTA was located within a LISA cluster, as identified by the smoothing methods in the 11 years between 2011 and 2021. Zero-inflated Poisson regression models are fitted when the outcome is a count with excess zeros. CAR models, which acknowledged the spatial dependency of the outcome within a Bayesian framework, were used for both the logistic as well as the ZIP regression models. The package CARBayes was used to fit the models in R.

## Results

**Fig. 2:**
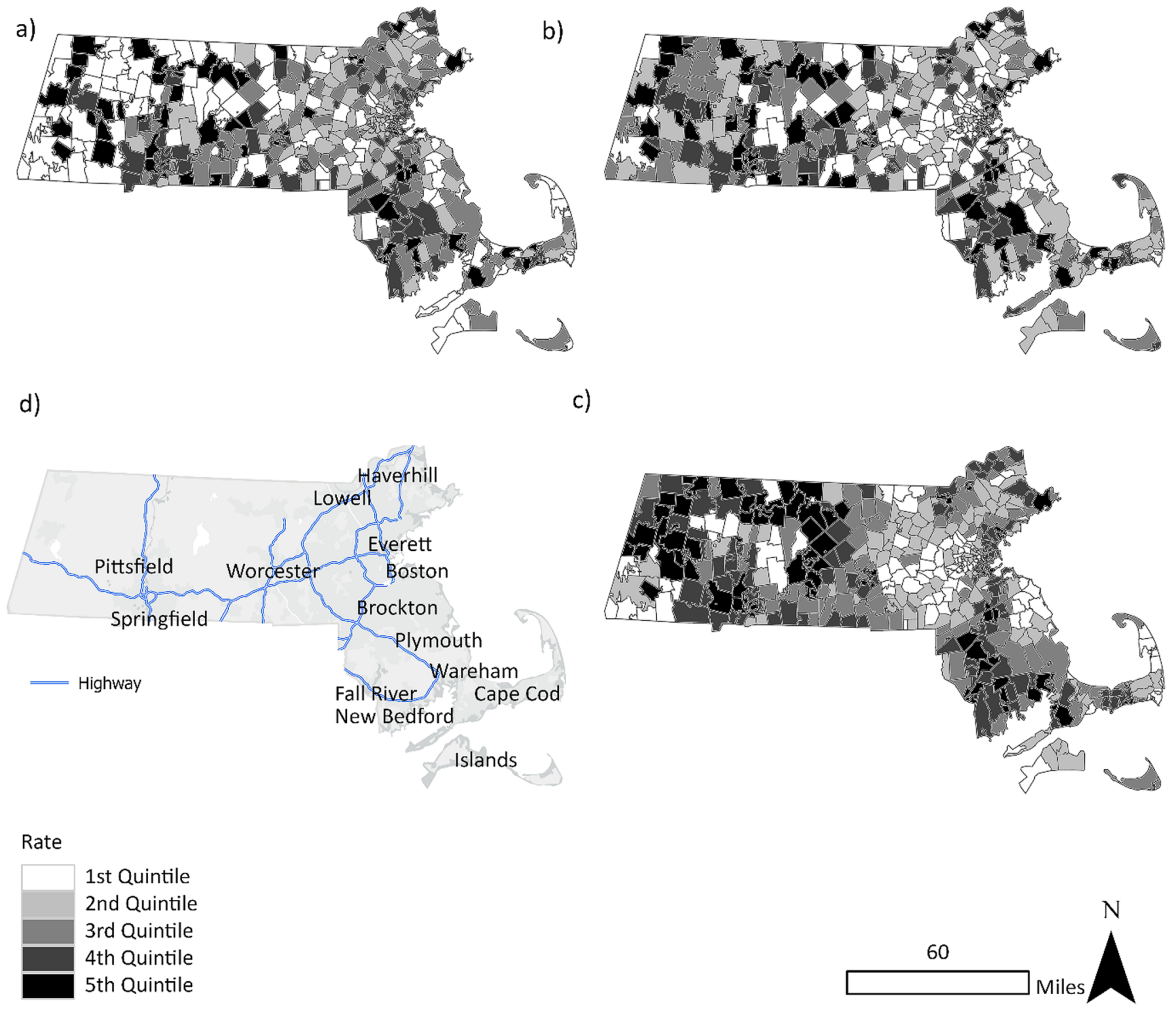
Fatal opioid overdose rate quintiles in 2021 in Massachusetts by ZIP Code Tabulation Area (ZCTA): 2(**a**) Raw rate; 2(**b**) Empirical Bayes (EB) smoothed rates; 2(**c**) Spatial EB smoothed rates 2(**d**) Reference map. This comparison of maps displaying raw and smoothed overdose rates highlights that identification of local high incidence clusters is easier in the EB and EB spatial maps than in the raw rate map’s patchwork of high and low values

**Fig. 3:**
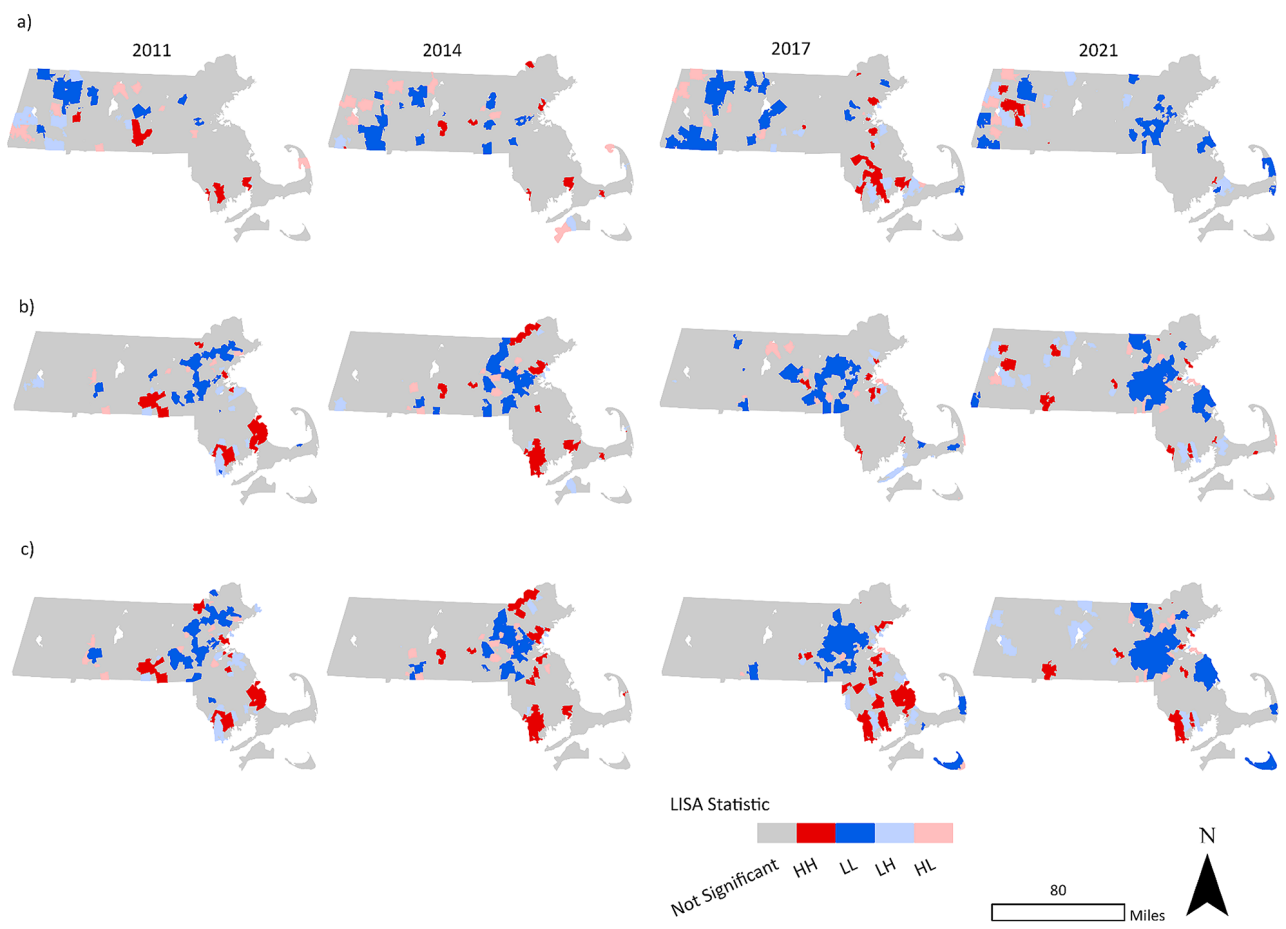
Fatal opioid overdose LISA clusters and outliers (*p* < 0.05) for 2011, 2014, 2017 and 2021: 3(**a**) Raw, 3(**b**) Empirical Bayes (EB) LISA, 3(**c**) EB smoothed death rates LISA by ZIP Code Tabulation Area (ZCTA), Massachusetts. LISA (Local Indicators of Spatial Association) analyses generate clusters of HH (high ZCTAs surrounded by ZCTAs with high rates), HL (high surrounded by low rates), LL (low surrounded by low rates), LH (low surrounded by high rates) or not significant

**Fig. 4:**
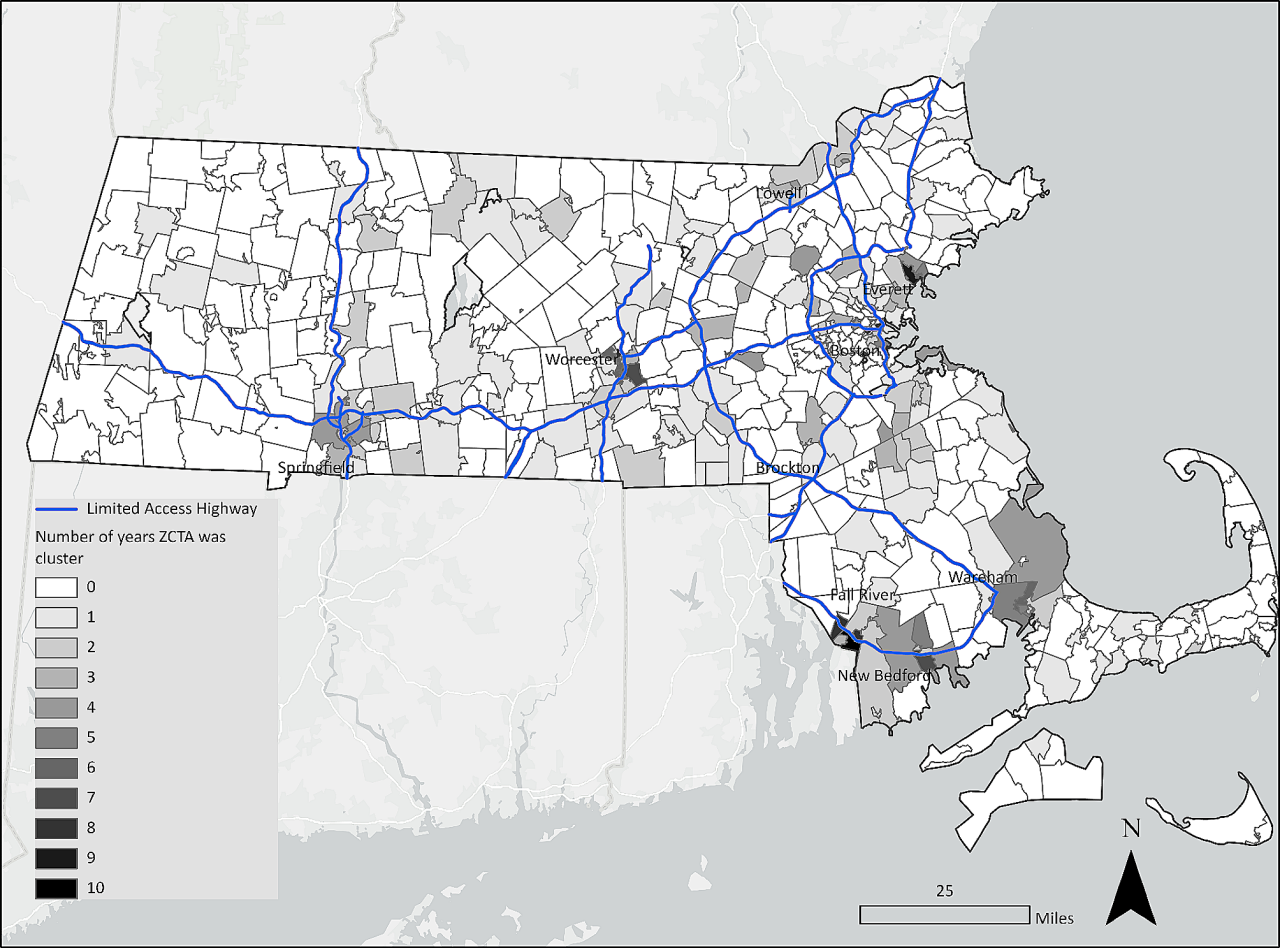
Number of times (i.e., years) the ZCTA was a HH or HL cluster between 2011–2021 using Empirical Bayes LISA (Local Indicators of Spatial Association)

The use of rate smoothing techniques facilitated the identification of high opioid overdose rate ZCTAs surrounding Pittsfield, Springfield, Fall River, and New Bedford, Massachusetts. Several of these locations had high overdose fatality rates in 2021 based on the smoothed data that were not easily identifiable in maps of the raw rates for the same year (Fig. 2). Figure 3 shows that the impact varied over time. For example, in 2011 the smoothed rates enabled identification of clusters in the Boston area, in addition to the area south of Worcester, Fall River, New Bedford, and Cape Cod (Fig. 3). Likewise, in 2014, using the raw rate alone resulted in fewer and more scattered clusters − 25 than the Empirical Bayes methods, which identified 45 and 53 ZCTAs as being part of LISA HH or HL clusters. The Empirical Bayes LISA and LISA of the Empirical Bayes rate methods also depicted more geographic variability, facilitating identification of clusters near Wareham in the southeast and Haverhill in the northeast. In 2017, the numbers of clusters identified based on the raw rates alone was comparable to those from the other methods − 36 versus 28 from the Empirical Bayes LISA method. The mapped results suggested that overdose deaths persisted in Worcester, Fall River, and Boston. By 2021, the number of ZCTAs identified within high overdose clusters using raw rates was much lower: 13. However, using Empirical Bayes LISA, the number of ZCTAs in clusters was 38. Figure 4 highlights locations of persistent HH and HL overdose clusters, with the most notable ongoing clusters in Worcester, New Bedford, Fall River, and Wareham. Many of these were not identified by the raw rate LISA, which only showed clusters in western Massachusetts in 2021 (Fig. 3).

We compared sociodemographic and built environment access variables in the statistically significant ZCTA LISA clusters, which illuminated important differences in sociodemographic factors. In ZCTAs that were always identified as being part of HH or HL LISA clusters, the mean percentages of the population living in poverty, and residents who were non-Hispanic Black or Hispanic, were higher than in ZCTAs that never appeared in fatal overdose clusters (Table 1). In terms of built environment variables, ZCTAs that were located within persistent clusters were closer to major roads and highway exits.

**Fig. 5:**
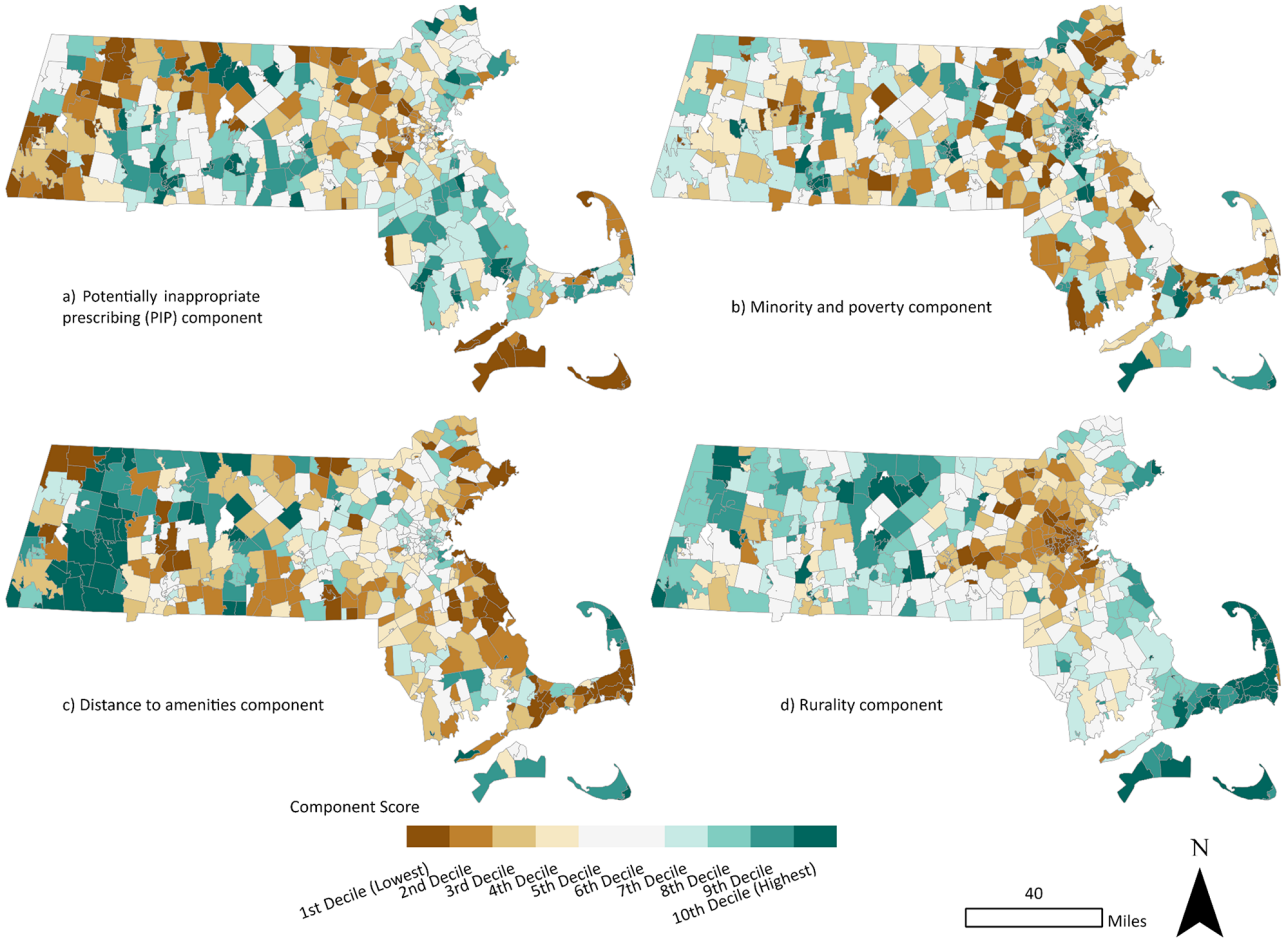
Principal Components Analysis scores mapped in deciles for Massachusetts by ZIP Code Tabulation Area (ZCTA): (**a**) RC1: potentially inappropriate prescribing (PIP); (**b**) RC2: Minority and poverty; (**c**) RC3: Distance to amenities (**d**) RC4: Rurality

The results of the PCA suggested that the four components we used in this analysis explained about 62% of the variance in the data (Table 2). The loadings on the first PCA component suggested that it was a measure of PIP because it had a positive loading (indicating positive correlation) with all PIP measures. This component had high scores in the central parts of the state along Interstate 90 and along Interstate 95 in the south (Fig. 5). The second component was correlated with poverty, non-Hispanic Black, American Indian and Alaska Native (AIAN) and Hispanic population percentages and we refer to it as the “minority and poverty” component. Locations that had high values for the minority and poverty component were found across Massachusetts in locations like Worcester, Springfield, New Bedford, Fall River, and in the southern area of Boston, within the Dorchester neighborhood (Fig. 5).

The third component, which we termed “distance to amenities,” was related to being relatively far from fast food restaurants, pharmacies, and gas stations. The ZCTAs with high values for the distance from amenities component were located not only in high poverty locations, such as urban neighborhoods in Boston, Worcester, New Bedford, and Springfield, but also in the more rural areas of Western Massachusetts, and on Cape Cod and the islands. Some high-income suburbs close to Boston like Carlisle, and Grafton (which is near Worcester) also had moderately high values for this component suggesting that it was not correlated with income (Fig. 5).

The fourth component, which we referred to as the “rurality” component, loaded positively on median age, and distance to a highway and major roads. As can be seen in Fig. 5, high scores for the rurality component were identified in western Massachusetts and Cape Cod, while low scores were observed in urban areas within and near Boston, Springfield, New Bedford, and Worcester.

The results of the CAR Bayesian logistic regressions for the outcome focused on whether a ZCTA was in a HH or HL cluster (derived from three different LISA statistics) for each year from 2011 to 2021 are shown in Table 3. We only included coefficients with statistically significant credible intervals from the CAR Bayesian regressions in Table 3 for brevity. Notably, the Empirical Bayes LISA, which was the most stable indicator of high overdose clusters observed in the maps, was the earliest to pick up any trends in the models over time. The LISA on Empirical Bayes smoothed rates also had similar results. LISA estimates of high overdose clusters from raw rates eventually also showed similarly significant credible intervals, but only after the trends had been observed in the other two model estimates.

The results of the CAR Bayes logistic model, predicting whether ZCTAs were located within a high overdose rate cluster using the Empirical Bayes LISA, suggested that for most years between 2011 and 2021, fatal opioid-involved overdose cluster ZCTAs were more likely to have a high minority and poverty component. The credible intervals for the minority and poverty component were lowest early in the study period in 2012 [2.13–4.47] and rose in 2021 [4.1–51.9] but they were always positive and significant. The PIP component was not significant in any of the models that were fitted. The distance to amenities and rural components, when significant, had a protective effect in that ZCTAs with high scores on these variables meaning that locations that were far from amenities were less likely to be in high overdose clusters. The odds ratio on the distance to amenities component varied from a higher range [0.29–0.89] in 2018 to a smaller range in in 2021 of [0.001–0.06]. The 95% credible intervals for the rurality component odds ratio varied from a lower range more recently in 2019 [0.05–0.67] to a wider range [0.07–0.99] in 2014.

The CAR Bayesian models predicting the number of times a ZCTA was in a high overdose rate cluster (Table 4) always had a positive and significant value within the credible interval for the minority and poverty component for the three models. This suggests that ZCTAs with high scores on this component in Worcester, Springfield, New Bedford, Brockton, Boston, and Fall River were more likely to have been a persistent overdose cluster over the 11-year period. The negative coefficients for the distance to amenities and rurality suggested that ZCTAs scoring high on distance to amenities and rurality were less likely to be located within persistent high overdose rate clusters (Table 4). The PIP component and the rurality component were not significant in any of the models.

## Discussion

In this study, we identified overdose clusters using rate smoothing techniques and found persistent and significant high incidence clusters in Massachusetts. We estimated PCA components to account for multicollinearity while incorporating opioid prescription, socio-environmental and built environment variables; and we acknowledged spatial autocorrelation in our models in the presence of spatial dependency in both the location of high overdose clusters and their persistence over the study period. Using PCA, we identified four unique components in Massachusetts that described ZCTA characteristics: PIP, Minority and Poverty, Distance to amenities, and Rurality. In the logistic regression models predicting if a ZCTA was part of a cluster at high risk of opioid-related overdose, we found that the minority and poverty factor was a significant predictor of a high overdose cluster in most years. In predicting the count of the number of times a ZCTA was part of a cluster, we found that the minority and poverty factor was again a significant predictor. Some recent studies have also noted the value of constructing variables that measure social disadvantage through the use of PCA [58, 59]. 

Zero inflation of disease rates is common in spatial epidemiological studies, which has resulted in several techniques to address this issue [31–39, 60]. We chose Empirical Bayes, and Empirical Bayes spatial smoothing methods, which reduced variance in the opioid fatality rates and thus resulting in better identification of high incidence clusters in ZCTAs that had relatively low population density. These rate smoothing techniques offer considerable promise in identifying regional clusters of persistent risk for fatal overdoses in Massachusetts earlier in the overdose epidemic than raw rates. Our application of the Empirical Bayes methods supported the use of this technique as a method to detect patterns at smaller spatial scales in which there was a mix of rural and urban areas [34]. Another recent study in Illinois noted the usefulness of EB clusters in a temporal study that could “serve as a proxy for pervasive risk” and “emerging risk” [61]. 

Our results may also indicate that minority and poverty presence in a ZCTA is significant in predicting high incidence clusters in Massachusetts. Several recent studies have also noted the role of poverty and race in predicting opioid overdose incidence in other states [16, 62–66]. The protective effect suggested by distance to amenities and rurality in the models suggests that measures of built environment and access to opioid supply may need to be incorporated into future analyses [16, 65]. It should also be noted that the negative association with rurality was no longer significant after 2019 which may be worth investigating in future work. A recent Massachusetts Department of Public Health report also noted this resurgence of opioid overdose prevalence in the most rural areas of the state in recent years [26]. 

The mapped LISA cluster estimates demonstrate that high overdose rate clusters that persisted over the years were comprised of higher percentages of non-Hispanic Black and AIAN populations, as well as Hispanic populations, and higher percentages of people living in poverty [66]. In 2011–2015, the overdose epidemic in Massachusetts and elsewhere was characterized in the media as affecting people who were middle income and non-Hispanic White as part of the “deaths of despair” narrative [67–71]. The role of poverty in opioid-involved overdoses in minoritized people who use drugs should be surveilled carefully as opioid overdoses continue to rise in these groups [72, 73, 74, 75].

Our findings have several limitations. For privacy reasons, the prescribing data (PIP) in this study were only available at the ZCTA scale and, therefore, our results are subject to ecological fallacy. Models that are at the individual level may show different relationships than our findings, which were aggregated to the ZCTA. Furthermore, we could not obtain PIP data for 2018–2021 and the PCA is based only on 2011–2017 data. However, the data for the earlier time periods were spatially clustered so it is likely that the later time periods will also show similar spatial and statistical correlations. The statistical associations that we identified may only be applicable to Massachusetts. Several laws and regulations were enacted in the state during the opioid overdose epidemic that may not be generalizable to other states. In 2019, for instance, the Governor of Massachusetts enacted a law that allowed no more than a seven-day supply of prescribed opioids (with certain exceptions), which, along with other policies, resulted in a notable decline in the number of opioid prescriptions dispensed [76].

## Conclusion

We employed a range of spatial analytical and statistical modeling approaches to better understand the opioid overdose crisis in Massachusetts and improve targeting of public health interventions. Smoothing methods allowed us to derive more stable estimates for opioid overdose rates and enhanced our understanding of the spatial clustering patterns related to fatal opioid-involved overdoses. We recommend using Empirical Bayes LISA when estimating clusters and Empirical Bayes smoothing for deriving rates especially for identifying locations at risk, particularly when tracking places that have a variety of population densities over space and time. These methods were able to identify clusters earlier in the epidemic than raw rates were able to in low population density locations. PCA facilitated a better understanding of unique community-level spatial and socioeconomic variables that may explain opioid-involved incidence. By using components that combined several socioeconomic, built environment, and prescription variables in the regression models, which also acknowledged spatial autocorrelation, we were able to characterize the significant contributing factors to opioid-related deaths in Massachusetts. Future research should investigate minority and poverty variables and their potential proxies. Many factors impact the cascade of opioid use, opioid use disorder, and opioid-involved overdose mortality, and by identifying factors at the beginning of the cascade, as we have done here, we can inform policies to intervene early in the cascade and prevent opioid-involved deaths.

**Table 1:** Mean (SD) of demographic and built environment variables by number of years in which a ZIP code tabulation area (ZCTA) was an empirical Bayes (EB) LISA HH (high surrounded by high) or HL (high surrounded by low)

Number of years ZCTA was in HH/HL cluster	ZCTA count	Percent Poverty	White Percent	Black Percent	Hispanic Percent	Asian Percent	American Indian/ Alaskan Native Percent	Male Percent
**0**	352	8.4 (8.8)	89.8 (12.3)	3.1 (7.6)	3.7 (4.5)	3.9 (6.3)	0.2 (0.6)	48.9 (7.5)
**1**	89	9.1 (7)	87.5 (12.3)	3.3 (4.3)	7 (11.2)	4.2 (7.2)	0.2 (0.3)	48.5 (5.9)
**2**	43	13.1 (10.8)	77.9 (21.4)	8.7 (14.5)	11.9 (15)	5.7 (7.9)	0.3 (0.3)	48.1 (2.4)
**3**	17	13.8 (8.7)	74.9 (18.1)	7.5 (10.2)	18.4 (22.2)	5.6 (5.3)	0.2 (0.1)	48.7 (2.7)
**4**	17	18.5 (14.6)	71.7 (19.4)	8.5 (9.4)	23.4 (24.4)	4.5 (4.9)	0.3 (0.3)	48.6 (1.9)
**5+**	17	25.6 (12.4)	67.5 (19.6)	11 (8.4)	22.1 (19.7)	3.9 (4.2)	0.3 (0.4)	48.2 (2.7)
**Number of years ZCTA was in HH/HL cluster**	ZCTA count	Median Age	Distance Gas station*	Distance fast food restaurant	Distance Pharmacy	Distance Highway exit	Distance major road	Gas station density**
**0**	352	44.2 (9.3)	573.2 (1515)	1951.3 (4292.3)	1755.1 (2935.9)	10082.5 (14839.4)	1263.7 (3730.2)	3505.4 (6574.6)
**1**	89	42 (7.3)	195.5 (954.6)	1009.1 (4995.6)	756 (1654.0)	7131.3 (12508.3)	1270.5 (5108.7)	4954.4 (7372.7)
**2**	43	39.6 (8.6)	48.7 (319.5)	153.6 (682.3)	418.5 (1518.7)	5434.0 (11840.9)	480.3 (1209)	7156.8 (6765.3)
**3**	17	38.6 (5.6)	0 (0)	0 (0)	55.5 (228.8)	2412 (3350.8)	714.6 (1614.1)	7551.9 (5384.3)
**4**	17	37.1 (7.1)	0 (0)	6.7 (27.8)	11 (45.4)	1388.8 (2133.1)	654.7 (1723.9)	8313.6 (6644.7)
**5+**	17	36.6 (6.9)	1.4 (5.7)	133.3 (527.6)	26.6 (83.1)	1521.1 (2399.4)	668.2 (1690.6)	8287.5 (5056.4)

*All distances in meters. Note that since distances are from the centroid of a ZCTA the presence of an amenity in a ZCTA will result in 0 m

**Density per square mile at the ZCTA level

*Abbreviations* SD = Standard Deviation, ZCTA = ZIP Code Tabulation Area

**Table 2:** Principal Components Analysis using a Varimax rotation: loadings of the opioid prescription, socioeconomic, and infrastructure variables for the first four components

	RC1: Potentially inappropriate prescribing (PIP)	RC2: Minority and Poverty	RC3: Distance to Amenities	RC4: Rurality
Percent living in Poverty		0.75		
White Percentage		-0.80		
Black Percentage		0.74		
AIAN Percentage		0.82		
Hispanic Percentage		0.40		
Asian Percentage				-0.58
Male Percentage			0.25	
Median Age	-0.38	-0.48		
Mean Distance to Gas Station			0.86	
Mean Distance to Fast food restaurant			0.61	0.67
Mean Distance to Pharmacy			0.87	
Mean Distance to Highway Exit				0.52
Mean Distance to Major Road				0.79
Mean Gas Station Density		0.49		
Opioid prescriptions without a pain diagnosis (Rate)	0.83			
>=3 cash purchases of opioid prescriptions (Rate)	0.95			
Poly-pharmacy opioid prescription fills (Rate)	0.98			
Multiple prescriber (Rate)	0.91			
Co-prescribing of benzodiazepines and opioids (Rate)	0.95			
Proportion of Variance in the data explained by component	24	16	11	10
Cumulative variance of data explained by component	24	40	52	62

*Abbreviations* RC = Rotated Component; AI/AN = American Indian/Alaskan Native

**Table 3:** Significant coefficients within the 95% credible interval (for CAR bayesian logistic model) associated with a ZCTA was a LISA (Local Indicators of Spatial Association) HH (high surrounded by high) or HL (high surrounded by low), Massachusetts, 2011–2021

	Raw Rate LISA				EB LISA				EB Smoothed Rate LISA			
	RC1	RC2	RC3	RC4	RC1	RC2	RC3	RC4	RC1	RC2	RC3	RC4
**2011**							-2.27	-1.50				
**2012**						0.35	-1.06			0.61	-1.66	
**2013**			-0.64			0.60	-2.70	-1.67		0.72	-2.54	
**2014**		0.38				1.50	-3.11	-3.04		0.66	-1.35	
**2015**		0.37				0.78	-1.41			0.94	-1.70	
**2016**						0.39	-1.01	-0.80		0.44	-1.03	
**2017**		0.51				1.02	-3.00	-1.77		0.95	-1.36	
**2018**		0.51	-1.68	-1.52		0.98	-3.86	-1.86		1.25	-3.75	-1.72
**2019**		1.06	-3.29	-1.38		1.23	-2.69	-1.60		1.62	-1.71	-0.76
**2020**		0.37				0.82				1.34	-1.46	
**2021**			0.43			0.71	-1.60			1.19	-2.04	
**N**	**535**											

RC1: Potentially inappropriate prescribing; RC2: Minority and Poverty; RC3: Distance to Amenities; RC4: Rurality

*Abbreviations* CAR = Conditional Autoregressive

**Table 4:** CAR bayesian zero inflated Poisson regression coefficients predicting the number of times the ZCTA was a LISA (Local Indicators of Spatial Association) HH (high surrounded by high) or HL (high surrounded by low), Massachusetts, 2011–2021

	LISA count			EB LISA count			EB Smoothed Rate LISA count		
	Mean	2.50%	97.50%	Mean	2.50%	97.50%	Mean	2.50%	97.50%
Intercept	-0.42	-0.83	0.02	-0.98	-1.34	0.51	-1.20	-1.43	-0.99
RC1	2.53	1.08	3.72	0.40	0.16	0.61	-0.11	-0.50	0.13
RC2	**0.26**	**0.11**	**0.41**	**0.53**	**0.39**	**0.67**	**0.44**	**0.30**	**0.58**
RC3	**-0.08**	**-0.33**	**-0.16**	**-1.11**	**-1.49**	**-0.75**	**-0.65**	**-1.03**	**-0.31**
RC4	0.08	-0.39	0.45	-0.17	-0.46	0.08	-0.24	-0.57	0.08

* Coefficients with significant 95% credible intervals are boldfaced

RC1: Potentially inappropriate prescribing; RC2: Minority and Poverty; RC3: Distance to Amenities; RC4: Rurality

*Abbreviations* CAR = Conditional Autoregressive

## Data Availability

The datasets used and/or analyzed during the current study are available from the corresponding author or the Massachusetts Department of Public Health upon request.
